# Electrochemical Sensor Based on Ni-Co Layered Double Hydroxide Hollow Nanostructures for Ultrasensitive Detection of Sumatriptan and Naproxen

**DOI:** 10.3390/bios12100872

**Published:** 2022-10-13

**Authors:** Hadi Beitollahi, Zahra Dourandish, Somayeh Tajik, Fatemeh Sharifi, Peyman Mohammadzadeh Jahani

**Affiliations:** 1 Environment Department, Institute of Science and High Technology and Environmental Sciences, Graduate University of Advanced Technology, Kerman 7631885356, Iran; 2 Department of Chemistry, Faculty of Science, Shahid Bahonar University of Kerman, Kerman 76175-133, Iran; 3 Research Center of Tropical and Infectious Diseases, Kerman University of Medical Sciences, Kerman 7616913555, Iran; 4 School of Medicine, Bam University of Medical Sciences, Bam 7661771967, Iran

**Keywords:** Ni-Co layered double hydroxide hollow nanostructures, screen-printed electrode, sumatriptan, naproxen

## Abstract

In this work, Ni-Co layered double hydroxide (Ni–Co LDH) hollow nanostructures were synthesized and characterized by X-ray diffraction (XRD), field emission-scanning electron microscopy (FE-SEM), and Fourier-transform infrared spectroscopy (FT-IR) techniques. A screen-printed electrode (SPE) surface was modified with as-fabricated Ni–Co LDHs to achieve a new sensing platform for determination of sumatriptan. The electrochemical behavior of the Ni–Co LDH-modified SPE (Ni-CO LDH/SPE) for sumatriptan determination was investigated using voltammetric methods. Compared with bare SPE, the presence of Ni-Co LDH was effective in the enhancement of electron transport rate between the electrode and analyte, as well as in the significant reduction of the overpotential of sumatriptan oxidation. Differential pulse voltammetry (DPV) was applied to perform a quantitative analysis of sumatriptan. The linearity range was found to be between 0.01 and 435.0 μM. The limits of detection (LOD) and sensitivity were 0.002 ± 0.0001 μM and 0.1017 ± 0.0001 μA/μM, respectively. In addition, the performance of the Ni-CO LDH/SPE for the determination of sumatriptan in the presence of naproxen was studied. Simultaneous analysis of sumatriptan with naproxen showed well-separated peaks leading to a quick and selective analysis of sumatriptan. Furthermore, the practical applicability of the prepared Ni-CO LDH/SPE sensor was examined in pharmaceutical and biological samples with satisfactory recovery results.

## 1. Introduction

Sumatripta (1-[3-(2-dimethylaminoethyl)-1H-indol-5-yl]-N-methylmethanesulfonamide) is one of the triptan drugs medically administrated to manage cluster headache and migraine [1,2]. Sumatriptan is a selective 5-hydroxytryptamine (5-HT_1B/1D_) receptor agonist [3]. Its binding with serotonin type-1D receptors leads to extensively dilated cranial vessel vasoconstriction, thus reducing migraine pain [4]. Reportedly, extra dose of triptans can be followed by numerous complications, some of which are neck tension, seizures, sleepiness, paralysis, hypertension, leg or arm swelling, and feeling tremor [5,6]. The physiological significance of sumatriptan makes it necessary to quantitatively measure sumatriptan in different specimens, particularly biological samples in the disciplines of clinical diagnosis, pharmacology, and the life sciences. Naproxen (6-methoxy-a-methyl-2-naphthalene acetic acid) is widely used as an anti-inflammatory drug to manage numerous medical conditions such as degenerative joint disorder, rheumatoid arthritis, primary dysmenorrhea, ankylosing spondylitis, and acute gout [7]. Nevertheless, extra and long-term naproxen use can develop some complications such as gastrointestinal hemorrhage, stomach ulcers, nephrotoxicity, elevated heart disease risk, and kidney dysfunction [8,9]. The medical importance of naproxen in humans highlights the necessity of access to an appropriate rapid, facile, and sensitive analytical approach. In addition, the mode of complementary action of these two drugs suggests their combined use to obtain more effective clinical outcomes in the treatment of acute migraine than both drugs alone. Hence, simultaneous detection of these drugs in biological fluids and pharmaceutical formulations is very important [10].

Many strategies, including spectrophotometry [11,12], capillary electrophoresis [13,14], high-performance liquid chromatography [15,16], liquid chromatography–mass spectroscopy [17], spectrofluorimetry [18], chemiluminescence [19], and electrochemical techniques [20,21,22] have been applied for determination of these compounds. However, the widespread application of some of these methods has been limited by their complex operation and high cost. Electrochemical determinations have been shown to be more appropriate for analyte analysis [23,24,25,26,27,28,29,30,31,32], owing to commendable merits such as cost-effectiveness, narrow LOD, higher sensitivities, wide potential window, short analysis time, and ease to renew the surface.

Screen-printing technology has proven its effectiveness in making electroanalytical platforms with tailored purposes, some of which are point-of-care (POC) tools in biomedicine [33,34,35], and portable sensing systems in food industries [36,37] and environmental pollutant detection [38,39,40]. SPEs are potent materials for electroanalytical (bio)sensors [41,42,43] owing to their inexpensiveness and easy production process, especially for the fabrication of transducers required for on-site one-point measurements. The miniaturized SPEs are appropriate for on-site measurements during real-time analysis, and require small amounts of reagents and samples.

The application of nanomaterials in various fields is increasing rapidly [44,45,46,47,48,49] and offers promising prospects. In recent years, the advances in nanotechnology have been helpful to produce sensitive and selective (bio)sensors [50,51,52,53,54,55,56]. A variety of nanomaterials, such as metal and metal oxides nanoparticles, and carbon nanostructures, have been employed to fabricate electrochemical (bio)sensing platforms [57,58,59,60,61,62,63,64,65], with diverse performances such as biomolecule labeling or immobilization, the electrochemical process catalysis, electron transfer enhancement, and serving as reactant [66].

Layered double hydroxides (LDHs) have recently spurred extensive interest owing to multiple specific merits such as a layered nature, huge surface area, adjustable structure, cost-effectiveness, and environmental friendliness [67,68,69]. The LDHs containing transition metals are of great significance for catalyst, energy storage, and sensing [70,71,72,73]. One of the strategies to enhancing their electrochemical performance is the design of tunable porous nanostructures or architecture of LDHs with huge surface area [74,75,76]. Hierarchical hollow structures (HHSs) with well-defined micro- or nanostructures, mesoporous pore-size distribution, huge surface area, more active sites, and satisfactory charge transfer could potentially promote the electrochemical behavior of LDHs [77].

Among these materials, nickel–cobalt layered double hydroxides (Ni-Co LDHs) have attracted particular interest in electrochemical sensors because of their low cost, good redox activity, and eco-friendly properties. They have an inverse spinel crystal structure, where Ni^2+^ is distributed at the octahedral sites and Co^3+^ is distributed at both tetrahedral and octahedral sites. This composition offers higher conductivity than that of Ni-Co LDH, which in turn enhances the electron transfer and improves the performance of electrochemical sensors [78,79].

In this research, a simple strategy was used to design an electrochemical sensing platform based on SPE modification with Ni-Co LDH which was employed for the determination of sumatriptan in the presence of naproxen. The Ni-Co LDH-modified SPE demonstrated better sensor features with a low LOD, high sensitivity, and wide linear range. The sumatriptan and naproxen sensing platform was characterized by the successful measurement of these analytes in sumatriptan tablets, naproxen tablets, and urine samples.

## 2. Experimental

### 2.1. Equipment

A Metrohm Autolab PGSTAT 320N Potentiostat/Galvanostat Analyzer (Utrecht, The Netherlands) with GPES (General Purpose Electrochemical System-version 4.9) software was applied for all electrochemical determinations at ambient temperature. The electrochemical sensors were prepared using DRP-110 SPEs (DropSens, Oviedo, Spain) featuring a silver pseudo-reference electrode, graphite working electrode, and graphite auxiliary electrode. A Metrohm 713 pH meter with glass electrode (Herisau, Switzerland) was recruited to determine and adjust the solution pH. Direct-Q^®^ 8 UV deionized water (Millipore, Darmstadt, Germany) was used to freshly prepare all solutions.

A Panalytical X’Pert Pro X-ray diffractometer (Almelo, The Netherlands) applying a Cu/Kα radiation (λ:1.54 Å) was used for XRD analysis, and a Bruker Tensor II spectrometer (Karlsruhe, Germany) was employed to capture the FT-IR spectra. An MIRA3 scanning electron microscope (Tescan, Brno, Czech Republic) was utilized for FE-SEM imaging.

### 2.2. Solvents and Chemicals

All solvents and chemicals applied in our protocol were of analytical grade and obtained from Merck and Sigma-Aldrich. Phosphate-buffered solution (PBS) was prepared using phosphoric acid and adjusted by NaOH to the desired pH value.

### 2.3. Synthesis of Ni-Co Layered Double Hydroxide Hollow Nanostructures

The Stöber method, with slight modification, was followed to prepare monodispersed silica (SiO_2_) spheres [80]. To this end, tetraethyl orthosilicate (TEOS) (6 mL) was dissolved drop by drop in a solution containing ethanol (75 mL), deionized water (10 mL), and aqueous ammonia (3.15 mL), followed by stirring at an ambient temperature for 5 h. The centrifugation was performed to extract the SiO_2_ spheres from the suspension, followed by rinsing by ethanol/deionized water. Finally, the obtained white precipitate was oven dried under vacuum condition at 65 °C for 12 h. Subsequently, the SiO_2_@Ni-Co LDH core–shell structures were produced by following the protocol reported by Li and coworkers [77]. In brief, 200 mg of pre-synthesized silica spheres were dispersed in 100 mL ethanol under ultrasonication for 1 h. Then, 3 mmol Ni(NO_3_)_2_.6H_2_O (2.5 g) and 1.5 mmol Co(NO_3_)_2_.6H_2_O (5 g) were dissolved into the above suspension. After that, 23 mL of aqueous ammonia solution was dispersed drop by drop in the suspension containing SiO_2_ spheres and metal salts while magnetically stirring for 1 h at room temperature. The co-precipitation process was carried out for deposition of hierarchical Ni-Co LDH nanosheets on SiO_2_ sphere surface. The centrifugation was performed to extract the resulting precipitate, followed by thoroughly rinsing by ethanol/deionized water. The obtained precipitate was oven-dried at 80 °C for 12 h. Finally, Ni-Co LDH hollow structures were formed after removal of silica cores by etching SiO_2_@Ni-Co LDH in 0.5 M KOH solution at for 1 h. The resulting product was centrifuged and rinsed thoroughly. The prepared Ni-Co LDH hollow structures were dried at 60 °C for 12 h.

### 2.4. Preparation of the Ni-Co LDH/SPE Sensor

A drop-casting technique was followed to fabricate the Ni-Co LDH/SPE. Thus, a certain amount of as-prepared Ni-Co LDH hollow nanostructures (1 mg) was subsequently dispersed in deionized water (1 mL) under 20 min ultrasonication. Then, the dispersed suspension (4 µL) was coated dropwise on the SPE surface and dried at the laboratory temperature.

### 2.5. Real Samples Preparation

Five sumatriptan tablets (labeled 50 mg of sumatriptan) purchased from a local pharmacy in Kerman (Iran) were pulverized together, and then 50 mg was dissolved in water (25 mL) under ultrasonication to prepare a sumatriptan solution. Then, variable volumes of diluted solution were poured into a 25 mL volumetric flask and brought to the final volume with PBS (pH = 7); the analyses were performed using a modified electrode.

Five naproxen tablets (labeled 500 mg of naproxen) purchased from a local pharmacy in Kerman (Iran) were pulverized together, and then 500 mg was dissolved in water (25 mL) under ultrasonication to prepare a naproxen solution. Then, variable volumes of diluted solution were poured into a 25 mL volumetric flask and brought to the final volume with PBS (pH = 7); the analyses were performed using modified electrode.

Moreover, 10 mL of refrigerated urine specimens were centrifuged at 1500 rpm for 20 min, followed by filtering the supernatant via 0.45 µm filter. Next, variable supernatant solution contents were placed in 25 mL volumetric flasks and diluted to the marks using PBS at the pH value of 7. Variable sumatriptan and naproxen contents were applied to spike the diluted urine specimens. At last, a standard addition method was followed to quantify the sumatriptan and naproxen.

## 3. Results and Discussion

### 3.1. Characterization of Ni-Co Layered Double Hydroxide Hollow Nanostructures

**Figure 1:**
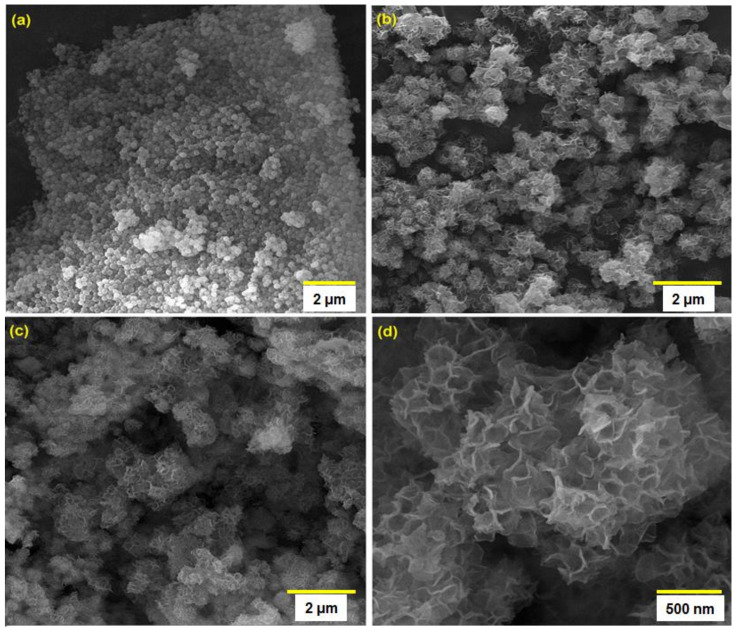
FE-SEM images of SiO_2_ spheres (**a**), SiO_2_@Ni-Co LDH core–shell structures (**b**), and Ni-Co LDH hollow structures (**c**,**d**).

The surface morphologies of SiO_2_ spheres, SiO_2_@Ni-Co LDH core–shell structures, and Ni-Co LDH hollow structures were explored using FE-SEM. Figure 1a shows the FE-SEM images of SiO_2_ spheres. The SiO_2_ spherical particles showed good monodispersity, with a uniform size of approximately 170 nm. According to the FE-SEM images captured from SiO_2_@Ni-Co LDH core–shell structures, it is clearly evident that, after the co-precipitation process, the hierarchical Ni-Co LDH nanosheets were well deposited on the surface of the silica spheres (Figure 1b). Subsequently, after KOH etching process to remove the SiO_2_ cores, the Ni-Co LDH hollow structures were obtained and showed an obvious hollow structure (Figure 1c,d).

**Figure 2:**
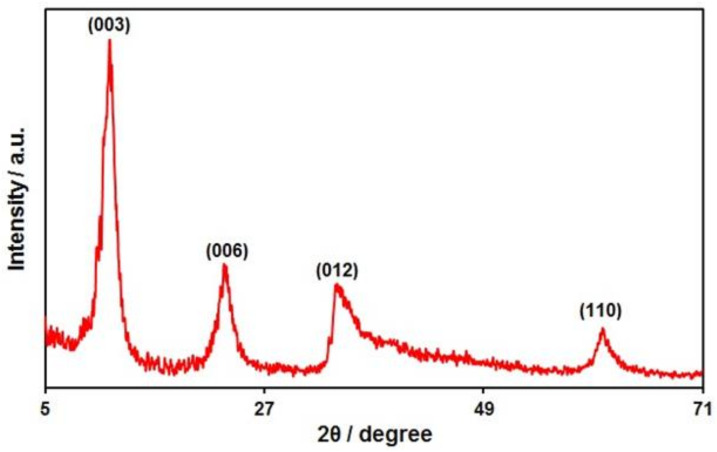
XRD pattern of Ni-Co LDH hollow structures.

The XRD pattern of Ni-Co LDH hollow structures is presented in Figure 2, showing the well-defined diffraction peaks observed at 2θ values of 11.4°, 23.0°, 34.2°, and 60.9° can be related to plane reflections of (003), (006), (012), and (110) for the hydrotalcite-like LDH phase. The XRD pattern of the synthesized sample is consistent with the XRD patterns reported in previous papers [81,82].

**Figure 3:**
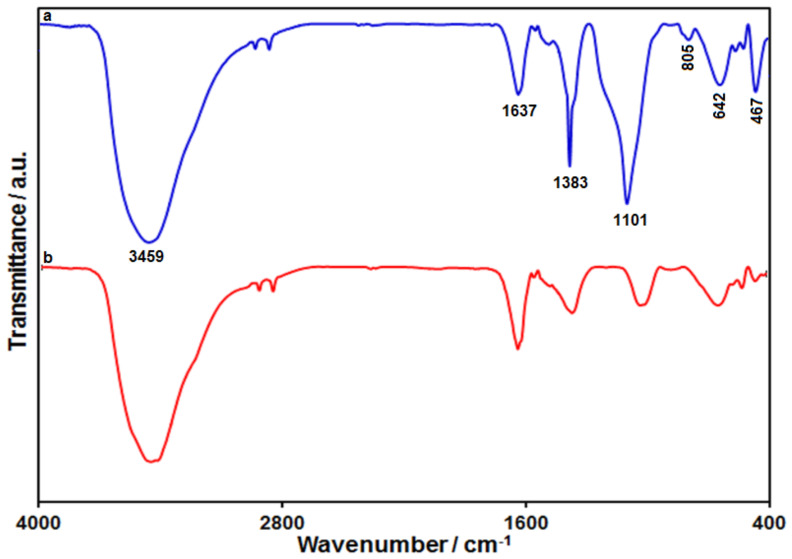
FT-IR spectra of (**a**) SiO_2_@Ni-Co LDH, and (**b**) Ni-Co LDH.

FT-IR spectroscopy is a well-equipped tool to study the functional groups of the prepared samples. Figure 3 depicts the FT-IR spectra of SiO_2_@Ni-Co LDH core–shell structures and Ni-Co LDH hollow structures. According to the FT-IR spectra captured from SiO_2_@Ni-Co LDH, the distinctive adsorption peaks of SiO_2_ were found at 467 cm^−1^, 805 cm^−1^, and 1101 cm^−1^, corresponding to the bending vibration of Si–O–Si, stretching vibration of Si–O–Si, and asymmetric stretching vibration of Si–O–Si [83]. Below, the existence of characteristic absorption bands of Ni-Co LDH is mentioned. The broad vibration of hydroxyl groups (O–H stretching) of water molecules in the interlayer space of LDH was confirmed at 3459 cm^−1^. The peak at 1637 cm^−1^ relates to the bending vibration of OH groups. The characteristic FT-IR band at 1383 cm^−1^ is generally assignable to the vibration of interlayer anions (CO_3_^2−^ and NO_3_^−^) [82,84]. In addition, the peak at 642 cm^−1^ relates to the characteristic absorption band of M–O (metal-oxygen) vibrations. According to the FT-IR spectra captured for Ni-Co LDH hollow structures, following the etching process, disappearing of Si–O–Si characteristic peaks highlighted the silica template removal.

### 3.2. Studying the Influence on the Structures on Voltammetric Detection of Sumatriptan Oxidation

**Figure 4:**
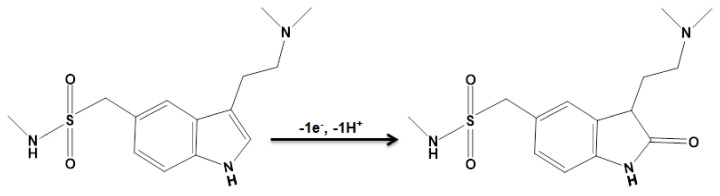
Proposed oxidation mechanism for sumatriptan.

**Figure 5:**
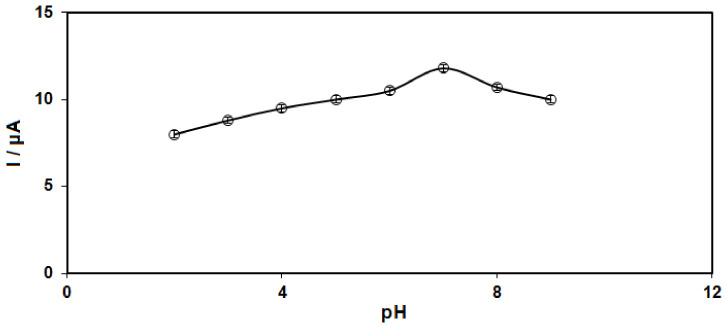
Plot of Ip vs. pH obtained from DPVs of Ni-Co LDH/SPE in a solution containing 100.0 μM of sumatriptan in 0.1 PBS with different pH (2.0, 3.0, 4.0, 5.0, 6.0, 7.0, 8.0, and 9.0).

The electrochemical response of sumatriptan oxidation in 0.1 M PBS adjusted to variable pH values (2.0 to 9.0) was explored to determine the influence of electrolyte solution pH. As shown in Figure 4, its electrochemical oxidation was dependent on the pH value of the solution, such that it reached a maximum with increasing pH up to 7.0 and then decreased with further pH values (Figure 5). Hence, the pH value of 7.0 was considered to be the optimum for subsequent electrochemical determinations.

**Figure 6:**
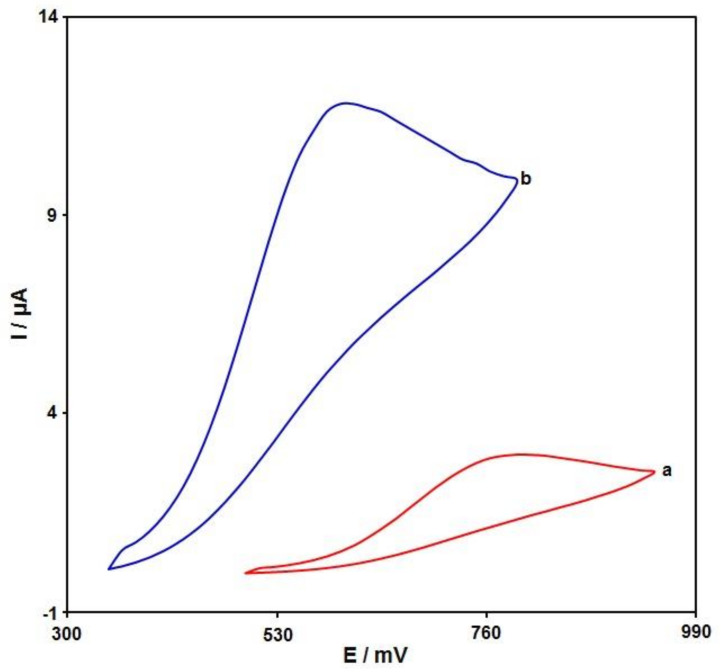
Cyclic voltammograms captured for oxidation of sumatriptan (100.0 μM) in PBS (0.1 M; pH = 7.0) on (a) unmodified SPE and (b) Ni-Co LDH/SPE with a scan rate of 50 mV/s.

Cyclic voltammetry (CV) was performed to clarify the electrochemical behavior of sumatriptan on unmodified (bare) and modified SPE surfaces. Figure 6 compares the bare SPE and Ni-Co LDH/SPE for 100.0 μM sumatriptan oxidation in 0.1 M PBS at the pH value of 7.0. The sumatriptan oxidation displayed a tiny and wide peak (2.9 μA) at the potential of 800 mV on the bare SPE surface. The Ni-Co LDH-modified SPE exhibited a shift in the peak current toward more negative potentials (610 mV) by raising the amount of current (11.8 μA). Such an improvement could have appeared because of the appreciable catalytic impact of Ni-Co LDH hollow nanostructures for sumatriptan oxidation.

### 3.3. Effect of Scan Rate

**Figure 7:**
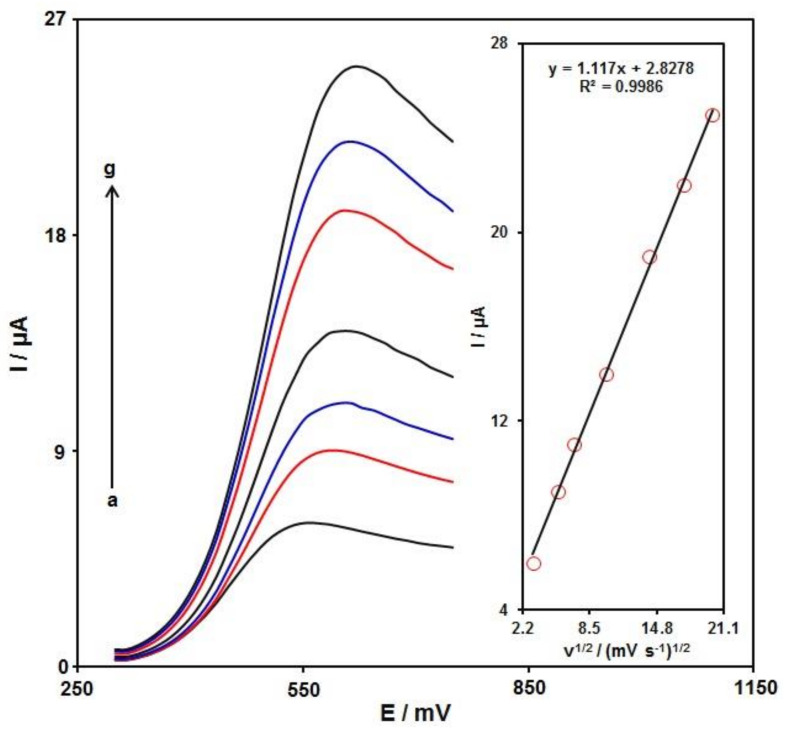
LSVs captured for the oxidation of sumatriptan (100.0 μM) on the Ni-Co LDH/SPE under variable scan rates (a–g: 10, 30, 50, 100, 200, 300, and 400 mV/s). Inset: correlation of Ipa with ʋ^1/2^.

The linear sweep voltammograms (LSVs) were recorded for the oxidation of sumatriptan (100.0 μM) on the Ni-Co LDH/SPE under variable scan rates (Figure 7). There was an apparent gradual elevation in the oxidation peak by raising scan rate ranging from 10 to 400 mV/s. As seen in Figure 7 (Inset), the anodic peak current (Ipa) had a linear association with the scan rate square root (ʋ^1/2^). The regression equation was obtained as Ipa (µA) = 1.117 ʋ^1/2^ (mV·s^−1^)^1/2^ + 2.8278 (R^2^ = 0.9986), indicating a controlled diffusion process of sumatriptan oxidation on the Ni-Co LDH/SPE.

**Figure 8:**
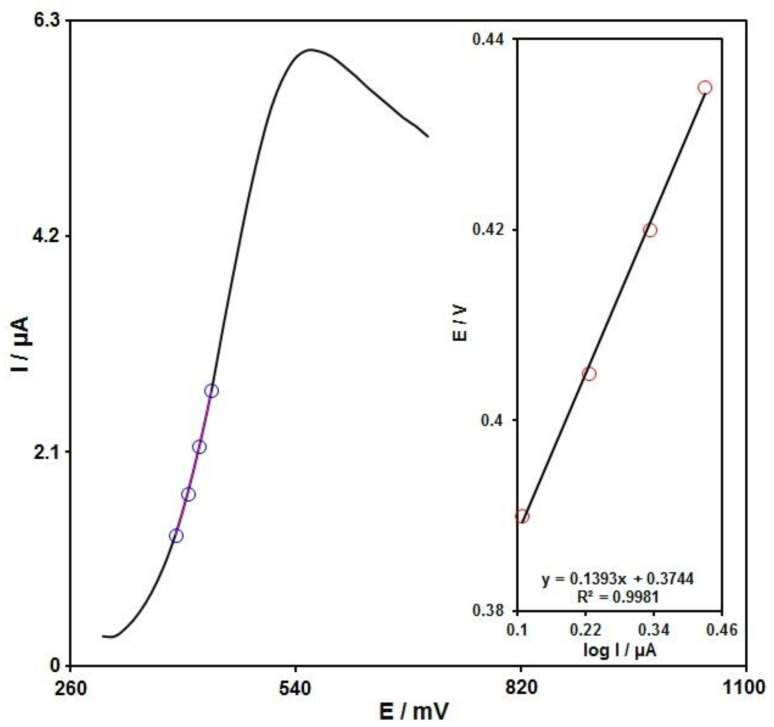
LSV for sumatriptan (100.0 μM) at the scan rate of 10 mV/s. Inset: The Tafel plot from the rising domain or the respective voltammogram.

A Tafel plot (Figure 8 (inset)) was achieved on the basis of data related to the rising domain of current–voltage curve at a low scan rate (10 mV/s) for sumatriptan (100.0 μM) to explore the rate-determining step. The linearity of the E vs. log I plot clarifies the involvement of electrode process kinetics. The slope from this plot could present the count of transferred electrons during the rate-determining step. On the basis of Figure 8 (inset), the Tafel slope was estimated to be 0.1393 V for the linear domain of the plot. The Tafel slope value revealed that the rate-limiting step was the one-electron transfer process considering a transfer coefficient (α) of 0.58.

### 3.4. Chronoamperometric Analysis

**Figure 9:**
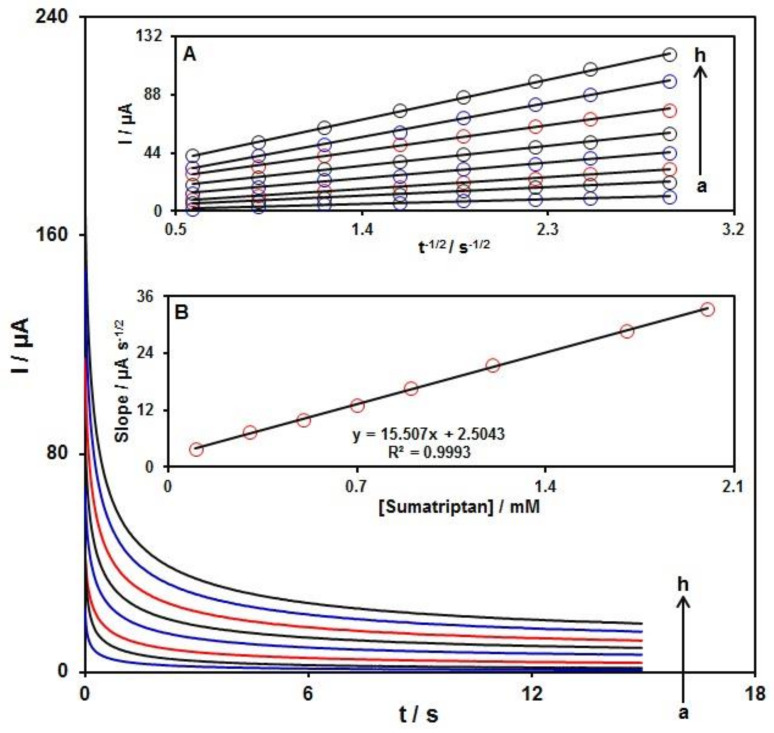
Chronoamperometric behavior of Ni-Co LDH/SPE in PBS (0.1 M; pH = 7.0) at potential of 660 mV for variable sumatriptan contents (a–h: 0.1, 0.3, 0.5, 0.7, 0.9, 1.2, 1.7, and 2.0 mM). Insets: (**A**) Plots of I vs. t^−1/2^ and (**B**) plots of the slopes from the straight lines vs. sumatriptan level.

Chronoamperometry was recruited to explore the sumatriptan catalytic oxidation on the Ni-Co LDH/SPE surface. Chronoamperometric analysis was performed for variable sumatriptan contents on Ni-Co LDH/SPE at the working electrode potential of 660 mV. The chronoamperograms captured for variable sumatriptan contents on the Ni-Co LDH/SPE are shown in Figure 9. Cottrell’s equation explains the current (I) for electrochemical reaction of an electroactive material with a D value (diffusion coefficient) under a mass transport limited condition. Figure 9A shows a linear relationship of the I value with t^−12^ for the oxidation of variable sumatriptan contents. The slopes from the obtained straight lines were plotted against variable sumatriptan contents (Figure 9B). The plotted slope and Cottrell equation estimated the D value to be 8.2 × 10^−5^ cm^2^/s for sumatriptan.

### 3.5. DPV Analysis of Sumatriptan

**Figure 10:**
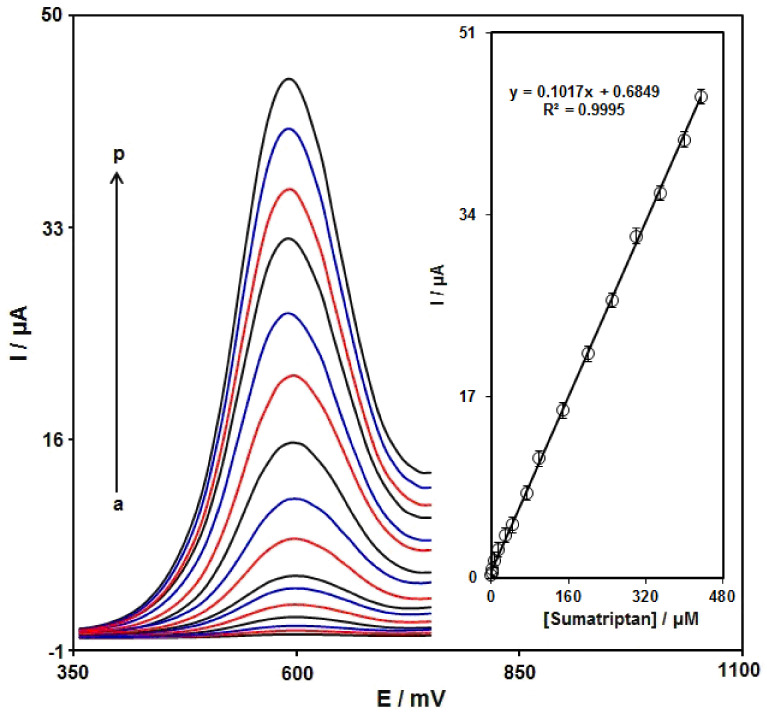
DPVs captured for the oxidation of variable sumatriptan contents on the Ni-Co LDH/SPE under variable contents (a–p: 0.01, 1.0, 2.5, 7.5, 15.0, 30.0, 45.0, 75.0, 100.0, 150.0, 200.0, 250.0, 300.0, 350.0, 400.0, and 435.0 μM). Inset: Calibration curve of voltammetric response (Ipa) against sumatriptan level.

DPV analysis was performed for variable sumatriptan contents to explore the linear dynamic range, LOD, and sensitivity of the Ni-Co LDH/SPE under optimized experimental circumstances (Figure 10). As expected, the elevation in sumatriptan level enhanced the peak current. Figure 10 (Inset) shows a linear proportionality of the oxidation peak currents to variable sumatriptan contents (0.01 μM to 435.0 μM) with a linear regression equation of Ipa (μA) = 0.1017 ± 0.0001 C_sumatriptan_ + 0.6849 (R^2^ = 0.9995), and a sensitivity of 0.1017 μA/μM. In the equations of LOD = 3S_b_/m and LOQ = 10S_b_/m, the S_b_ is the standard deviation of the response for blank solution, and m is the slope from the standard graph. The LOD and LOQ were estimated at 0.002 ± 0.0001 and 0.007 ± 0.0001 μM for sumatriptan determination on Ni-Co LDH/SPE.

Table 1 compares the efficiency of the sumatriptan sensor prepared by the Ni-Co LDH-modified SPE and other reported works.

### 3.6. DPV Analysis of Sumatriptan in the Presence of Naproxen

**Figure 11:**
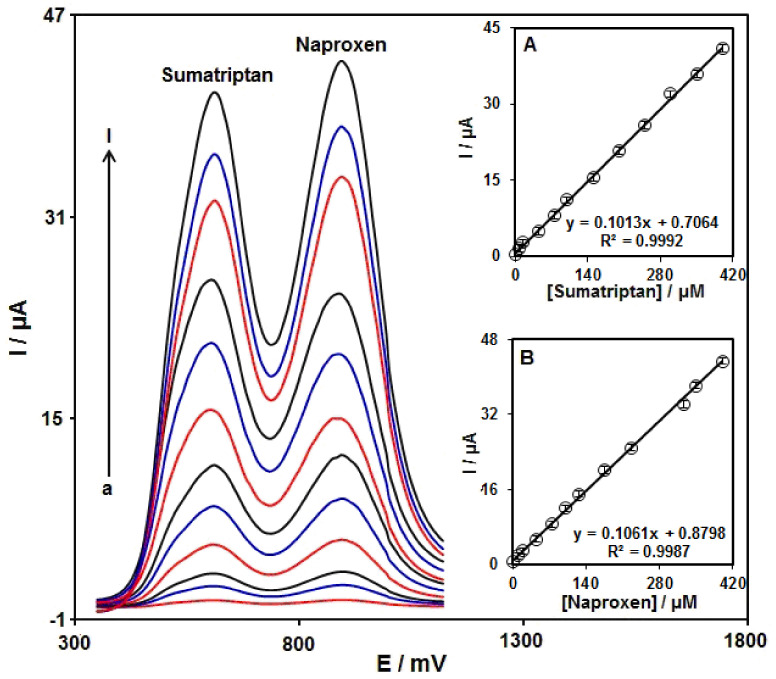
DPVs of Ni-Co LDH/SPE in 0.1 M PBS (pH 7.0) with various concentrations of sumatriptan (a–l: 1.0, 7.5, 15.0, 45.0, 75.0, 100.0, 150.0, 200.0, 250.0, 300.0, 350.0, and 400.0 μM) and naproxen (a–l: 1.0, 10.0, 20.0, 45.0, 75.0, 100.0, 125.0, 175.0, 225.0, 325.0, 350.0, and 400.0 μM). Insets: (**A**) The plot of peak current versus sumatriptan concentration, (**B**) the plot of peak current versus naproxen concentration.

To confirm the ability of Ni-Co LDH/SPE for codetection of sumatriptan and naproxen, the electrochemical responses of these analytes were detected by simultaneously changing the concentration of both analytes in PBS at pH 7.0. As seen in Figure 11, with a concurrent change in their concentrations, two noninterference peaks were found on DPV curves. The peak currents of both sumatriptan and naproxen oxidation displayed a linear elevation with the respective concentrations (sumatriptan concentration range between 1.0 μM and 400.0 μM, and naproxen concentration range between 1.0 μM and 400.0 μM) (Figure 11A,B). The intensity of peak current showed good linearity with the target concentration change, highlighting the possibility of detecting sumatriptan and naproxen in the blended solution.

### 3.7. Repeatability, Reproducibility, and Stability

The Ni-Co LDH/SPE was examined for repeatability through the measurement of the response of 40.0 μM sumatriptan on the surface of the same electrode 15 times. The relative standard deviation (RSD) of 3.9% for the current response of sumatriptan demonstrated the good repeatability of the proposed electrode.

To test the reproducibility, five Ni-Co LDH/SPE produced using the same procedures were applied to measure 40.0 µM sumatriptan under identical circumstances; the obtained RSD of 3.5% demonstrated commendable reproducibility.

To test the Ni-Co LDH/SPE stability, the current responses of sumatriptan were measured following 14 day storage of the sensor at ambient temperature. The decrease in peak current of sumatriptan to 4.2% of its original response demonstrated appreciable stability.

### 3.8. Selectivity Studies

The effects of some organic and inorganic species which commonly existed in pharmaceuticals and biological samples were examined on the analytical response of the proposed sensor (Ni-Co LDH/SPE). Therefore, a 50.0 µM solution of sumatriptan in the supporting electrolyte (PBS) was prepared. Various amounts of the interfering species were added to the sumatriptan solution. The voltammogram (DPV) of the sample was recorded in the presence of interfering species. The tolerance limit was defined as the maximum concentration of the interfering substance that caused an approximately ±5% relative error in the determination. The results revealed that 500-fold concentrations of Na^+^, Mg^2+^, Ca^2+^, NH_4_^+^, and SO_4_^2-^, 300-fold concentrations of fructose, glucose, and lactose, 100-fold concentrations of histidine, phenyl alanine, methionine, and cysteine, and 20-fold concentrations of levodopa and uric acid did not show interference in determination (Table S1, Supplementary Materials). These results confirmed the suitable selectivity of the proposed sensor for determination.

### 3.9. Analysis of Real Specimens

The practical applicability of Ni-Co LDH/SPE was tested by sensing sumatriptan and naproxen in sumatriptan tablets, naproxen tablets, and urine specimens using the DPV procedure and a standard addition method, the results of which can be seen in Table 2. The recovery rate was between 96.4% and 102.5%, and all RSD values were ≤3.6%. According to the experimental results, the Ni-Co LDH/SPE sensor possesses a high potential for practical applicability.

## 4. Conclusions

In this work, we reported the sensing application of Ni-Co LDH hollow nanostructures for electrochemical determination of sumatriptan. The sensing platform was fabricated via drop casting of a Ni-Co LDH hollow nanostructures dispersion on bare SPE. The electrochemical studies demonstrated efficient electrocatalytic activity of Ni-Co LDH hollow nanostructure-modified SPE for sensitive detection of sumatriptan. DPV findings showed an increase in the anodic peak currents with elevating sumatriptan contents (0.01–435.0 µM), with an LOD of 0.002 ± 0.0001 μM. Furthermore, for sensing sumatriptan in the presence of naproxen, the obtained voltammograms exhibited a desirable peak separation of about 300 mV potential differences. Moreover, the prepared sensor (Ni-Co LDH/SPE) was efficiently applied to detect sumatriptan and naproxen in in pharmaceutical and biological samples.

## Figures and Tables

**Table 1:** Comparison of the efficiency of the Ni-Co LDH/SPE sensor with other reported modified electrodes for sumatriptan determination.

Electrochemical Sensor	Electrochemical Method	Linear Range	LOD	Ref.
CuO/SPE	DPV	0.33–3.54 μM	0.066 μM	[4]
Cu nanoparticles (NPs)/poly-melamine/glassy carbon electrode	DPV	0.08–0.58 and 0.58–6.5 μM	0.025 μM	[85]
Multiwalled carbon nanotube (MWCNTs)decorated with silver NPs/pyrolytic graphite electrode	CV	0.08–100.0 μM	0.04 μM	[86]
MWCNTs and cobalt methyl-salophen complex/carbon paste electrode	DPV	1.0–1000.0 μM	0.3 μM	[21]
MWCNTs and polypyrrole doped with new coccine/glassy carbon electrode	LSV	0.02–10.0 μM	0.006 μM	[20]
Overoxidized poly(*p-*aminophenol) modified glassy carbon electrode	Square wave voltammetry	1.0–100.0 μM	0.294 μM	[87]
Ni-Co LDH/SPE	DPV	0.01–435.0 μM	0.002 μM	This work

**Table 2:** Voltammetric sensing of sumatriptan and naproxen in real specimens using Ni-Co LDH/SPE. All concentrations are in µA (*n* = 3).

Sample	Spiked (μM)		Found (μM)		Recovery (%)		RSD (%)	
	Sumatriptan	Naproxen	Sumatriptan	Naproxen	Sumatriptan	Naproxen	Sumatriptan	Naproxen
Sumatriptan Tablet	0	0	4.0	-	-	-	3.3	-
	1.0	4.0	4.9	4.1	98.0	102.5	1.9	2.3
	3.0	6.0	7.1	5.8	101.4	96.7	2.8	3.0
Naproxen Tablet	0	0	-	5.0	-	-	-	2.9
	5.0	1.0	5.1	5.9	102.0	98.3	3.0	2.2
	7.0	3.0	6.9	8.3	98.6	103.7	1.8	3.6
Urine	0	0	-	-	-	-	-	-
	4.5	5.5	4.6	5.3	102.2	96.4	2.5	2.8
	6.5	7.5	6.3	7.6	96.9	101.3	3.1	1.9

## Data Availability

All the data are presented in the manuscript.

## Supplementary Materials

The following supporting information can be downloaded at: https://www.mdpi.com/article/10.3390/bios12100872/s1, Table S1: Selectivity results for 50.0 µM sumatriptan in the presence of other interferences.
