# Supplementary material for: Electrochemical Sensor Based on Ni-Co Layered Double Hydroxide Hollow Nanostructures for Ultrasensitive Detection of Sumatriptan and Naproxen
Source: Biosensors (Basel). 2022 Oct 13;12(10):872. doi: 10.3390/bios12100872 (PMC9599541; doi:10.3390/bios12100872)
Supplement: Supplementary file 1 [file biosensors-12-00872-s001.zip › biosensors-1909519-supplementary-done.pdf]

biosensors

MDPI

Electrochemical Sensor Based on Ni–Co Layered Double Hydroxide Hollow Nanostructures for Ultrasensitive Detection of Sumatriptan and Naproxen

Hadi Beitollahi [1,*], Zahra Dourandish [2], Somayeh Tajik [3], Fatemeh Sharifi [3] and Peyman Mohammadzadeh Jahani [4]

1 Environment Department, Institute of Science and High Technology and Environmental Sciences, Graduate University of Advanced Technology, Kerman, 7631885356, Iran
2 Department of Chemistry, Faculty of Science, Shahid Bahonar University of Kerman, Kerman 76175-133, Iran
3 Research Center of Tropical and Infectious Diseases, Kerman University of Medical Sciences, Kerman 7616913555, Iran
4 School of Medicine, Bam University of Medical Sciences, Bam 7661771967, Iran
* Correspondence: h.beitollahi@yahoo.com

Table S1. Selectivity results for 50.0 μM sumatriptan in the presence of other interferences.

Species	Current (μA)
50.0 μM sumatriptan	5.5
50.0 μM sumatriptan+500-fold of Na^+	5.55
50.0 μM sumatriptan+500-fold of Mg^{2+}	5.57
50.0 μM sumatriptan+500-fold of Ca^{2+}	5.47
50.0 μM sumatriptan+500-fold of NH_4^+	5.53
50.0 μM sumatriptan+300-fold of fructose	5.60
50.0 μM sumatriptan+300-fold of glucose	5.63
50.0 μM sumatriptan+300-fold of lactose	5.61
50.0 μM sumatriptan+100-fold of hystidine	5.42
50.0 μM sumatriptan+100-fold of phenyl alanine	5.44
50.0 μM sumatriptan+100-fold of methionine	5.43
50.0 μM sumatriptan+100-fold of cysteine	5.40
50.0 μM sumatriptan+20-fold of levodopa	5.60
50.0 μM sumatriptan+20-fold of uric acid	5.65
